# Effects of ticagrelor on the pharmacokinetics of rivaroxaban in rats

**DOI:** 10.1080/13880209.2020.1785510

**Published:** 2020-07-07

**Authors:** Jia Chong, Hao Chen, Dapeng Dai, Shuanghu Wang, Quan Zhou, Junpeng Liu, You Lü, Hualan Wu, Minghui Du, Feifei Chen, Hui Jiang, Yunfang Zhou, Jiefu Yang

**Affiliations:** a Division of Cardiology, Internal Medicine Department, Beijing Hospital, Beijing, P.R. China; b Beijing Institute of Geriatrics, Beijing Hospital, Beijing, P.R. China; c The Laboratory of Clinical Pharmacy, The People’s Hospital of Lishui, Lishui, P.R. China

**Keywords:** Anticoagulation, atrial fibrillation, drug–drug interaction, rat liver microsomes, UPLC-MS/MS

## Abstract

**Context:**

Rivaroxaban and ticagrelor are two common drugs for the treatment of atrial fibrillation and acute coronary syndrome. However, the drug–drug interaction between them is still unknown.

**Objective:**

To investigate the effects of ticagrelor on the pharmacokinetics of rivaroxaban in rats both *in vivo* and *in vitro*.

**Materials and methods:**

A sensitive and reliable UPLC-MS/MS method was developed for the determination of rivaroxaban in rat plasma. Ten Sprague-Dawley rats were randomly divided into ticagrelor pre-treated group (10 mg/kg/day for 14 days) and control group. The pharmacokinetics of orally administered rivaroxaban (10 mg/kg, single dose) with or without ticagrelor pre-treatment was investigated with developed UPLC-MS/MS method. Additionally, Sprague-Dawley rat liver microsomes were also used to investigate the drug–drug interaction between these two drugs *in vitro*.

**Results:**

The *C*_max_ (221.34 ± 53.33 *vs.* 691.18 ± 238.31 ng/mL) and the AUC_(0–t)_ (1060.97 ± 291.21 *vs.* 3483.03 ± 753.83 μg·h/L) of rivaroxaban increased significantly (*p* < 0.05) with ticagrelor pre-treatment. The MRT_(0–∞)_ of rivaroxaban increased from 4.41 ± 0.79 to 5.97 ± 1.11 h, while the intrinsic clearance decreased from 9.93 ± 2.55 to 2.89 ± 0.63 L/h/kg (both *p* < 0.05) after pre-treated with ticagrelor. Enzyme kinetic study indicated that ticagrelor decreased rivaroxaban metabolic clearance with the IC_50_ value of 14.04 μmol/L.

**Conclusions:**

Our *in vivo* and *in vitro* results demonstrated that there is a drug–drug interaction between ticagrelor and rivaroxaban in rats. Further studies need to be carried out to verify whether similar interactions truly apply in humans and whether these interactions have clinical significance.

## Introduction

For decades, anticoagulation with vitamin K antagonists (VKAs) has been the only choice in the treatment of atrial fibrillation (AF) and other thromboembolic diseases. Recently, direct oral anticoagulants (DOACs), including dabigatran, rivaroxaban, apixaban and edoxaban, have emerged as alternatives to VKAs, due to their more predictable pharmacokinetic and pharmacodynamic profiles and less food and drug interactions (Heidbuchel et al. 2015; Steffel et al. 2018). Rivaroxaban, as a direct Factor Xa inhibitor, has been shown to be effective and well tolerated in the long-term treatment of non-valvular atrial fibrillation (NVAF) (Patel et al. 2011). In phase I trials, rivaroxaban was rapidly absorbed with the time to peak plasma concentration (*T*_max_) of 1.5–4 h, the area under the concentration (AUC) and the maximum concentration (*C*_max_) were dose dependent for single daily doses and at steady state after multiple daily dosing (Abrams and Emerson 2009). About one-third of the dose is removed in the unchanged form in the urine (mostly through active renal secretion by P-glycoprotein). About two-thirds is degraded by means of oxidative degradation and hydrolysis. Rivaroxaban is metabolized by cytochrome P450 enzymes (CYP3A4/5, CYP2J2) and CYP-independent ways. CYP3A4 is involved in about 18% and CYP2J2 in about 14% of total rivaroxaban elimination. Rivaroxaban is metabolized to M2 by CYP3A4, and M2 is transformed to more stable M1. The main metabolites of rivaroxaban are M1 and M4 (Kvasnicka et al. 2017). Potential drug–drug interactions may occur between rivaroxaban and drugs metabolized by CYP3A4. It has been reported that CYP3A4 and P-glycoprotein (P-gp) inhibitors ketoconazole, itraconazole, ritonavir, clarithromycin can increase the systemic exposure of rivaroxaban (Walenga and Adiguzel 2010), while some CYP3A4 and P-gp inducers, such as rifampicin and carbamazepine, can accelerate the intrinsic clearance value of rivaroxaban (Wessler et al. 2013). In one study, co-administration of ketoconazole caused a 2.6-fold increase in the mean AUC value of rivaroxaban, a 1.7-fold increase in *C*_max_ value, and accomplished with increased bleeding risk (Mueck et al. 2013). Similarly, increased rivaroxaban plasma concentrations were also reported when co-administrated it with verapamil and diltiazem (Kim et al. 2019). Ritonavir, a strong dual inhibitor of CYP3A4 and P-gp, was reported to increase the rivaroxaban concentration to a clinically relevant degree (Rathbun and Liedtke 2011). On the other hand, one CYP3A4 inducer rifampicin could cause a 50% reduction in the systemic exposure of rivaroxaban (Eriksson et al. 2009; Altena et al. 2014). It is suggested that co-administration of rivaroxaban with strong CYP3A4 or P-gp inhibitors, such as ketoconazole or ritonavir, should be avoided.

P2Y12 receptor antagonists represent the cornerstone drug for acute coronary syndrome (ACS) treatment. Unlike clopidogrel and prasugrel, the new generation P2Y12 inhibitor ticagrelor is reversible and directly acting, which does not require metabolic activation and has more predictable efficiency (Hulot et al. 2006; Giezen et al. 2009; Sorich et al. 2014; Tresukosol et al. 2014). Compared with clopidogrel, ticagrelor significantly reduced the incidence of the composite end point of cardiovascular death, MI or stroke in patients with ACS in PLATO study (Wallentin et al. 2009). Current guidelines recommend that ticagrelor is more favourable than clopidogrel in patients with ACS who receive medical therapy or coronary intervention (Windecker et al. 2014; Levine et al. 2016).

Ticagrelor is mostly metabolized by CYP3A4. It is not only a substrate but also an inhibitor of CYP3A4 (Teng et al. 2010). It has been shown that ticagrelor could significantly increase the systemic exposure of simvastatin and atorvastatin (Teng et al. 2013; Kido et al. 2015; Banakh et al. 2017). Similarly, ticagrelor is also a substrate and inhibitor of P-gp. Concomitant use of ticagrelor and digoxin increased digoxin *C*_max_ and AUC values by 75% and 28%, respectively (Teng and Butler 2013).

Approximately 1 in 5 persons with atrial fibrillation will undergo percutaneous coronary intervention or have an acute coronary syndrome, thus there is a high possibility of administration of rivaroxaban with ticagrelor together. Careful consideration of antithrombotic therapy and balancing bleeding risk is crucial. However, no reports have been concerned with the potential drug–drug interactions between ticagrelor and rivaroxaban. In this study, we developed an UPLC–MS/MS method for the quantification of rivaroxaban and use it for the evaluation of drug–drug interaction between ticagrelor and rivaroxaban in rats both *in vivo* and *in vitro*.

## Materials and methods

### Chemicals and reagents

**Figure 1.**
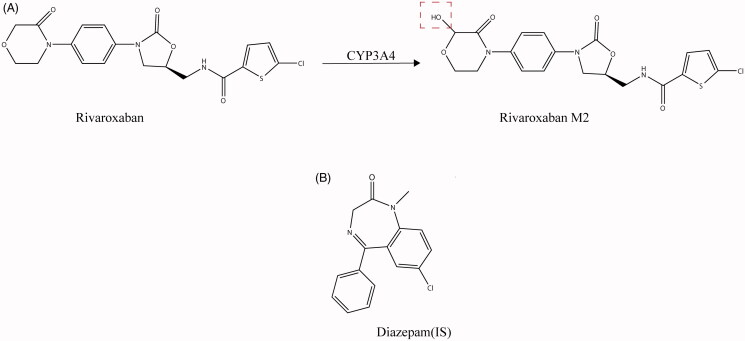
The chemical structures of rivaroxaban and its metabolite M2 (A) and IS. (B).

Rivaroxaban (purity >99%) and diazepam (purity >98%) were both purchased from J&K Scientific Ltd. (Beijing, China) (Figure 1). Rivaroxaban metabolite M2 (Lot No. 3530-047A1) was purchased from TLC Pharmaceutical Standards Ltd. (Newmarket, Ontario). Ticagrelor (purity > 98%) was purchased from Aladdin (Aladdin, Shanghai, China). Reduced nicotinamide adenine dinucleotide phosphate (NADPH) was obtained from Roche Pharmaceuticals Ltd. (Basel, Switzerland). Acetonitrile and methanol were obtained from Fisher Scientific Co. (Fair Lawn, NJ, USA). Formic acid was got from Sigma-Aldrich (St. Louis, MO, USA). Ultrapure water was obtained from a Milli-Q water purification system (Millipore, Billerica, MA, USA). All other chemicals were of analytical grade or better.

### Instruments and conditions

Chromatographic separation was performed using the Acquity UPLC system (Waters Corp., Milford, MA, USA) and ACQUITY UPLC HSS T3 column (2.1 × 100 mm, 1.8 μm) at 40 °C. The mobile phase comprising acetonitrile (A) and 0.1% formic acid water (B) was set with a flow rate of 0.40 mL/min. The gradient programme was applied as follows: 0–0.5 min, 30% A; 0.5–1 min, 30–95% A; 1–2 min, 95% A; 2–2.6 min, 95% A; 2.5–2.6 min, 95–30% A; 2.6–3 min, 30% A. The total time required for analysis was 3 min. The sample manager underwent a strong wash (methanol water, 50/50, V/V) and a weak wash (methanol water, 10/90, V/V) after each injection.

**Figure 2.**
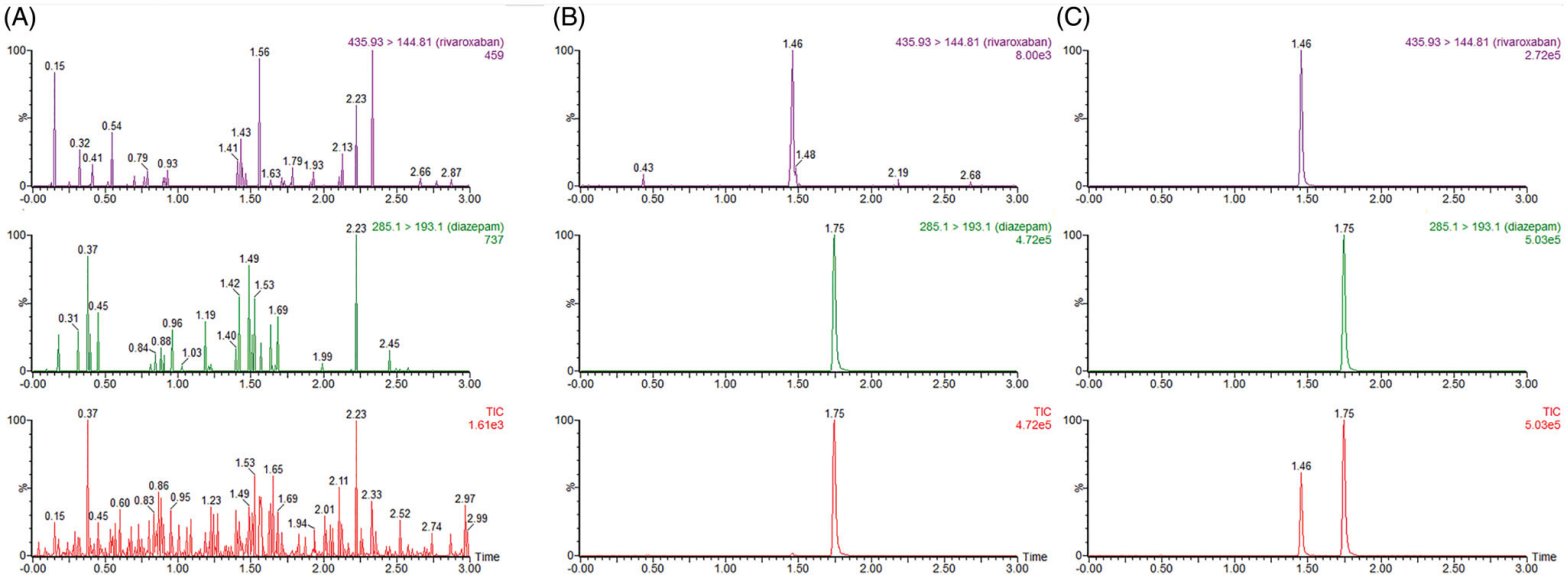
The MRM chromatograms of rivaroxaban and IS. (1) Blank plasma sample, (2) blank plasma sample with rivaroxaban (LLOQ) and IS., and (3) plasma sample after 1 hour of oral administration of rivaroxaban.

XEVO TQD triple quadrupole mass spectrometer was equipped with electrospray ionization (ESI), and multiple-reaction monitoring (MRM) mode was selected for quantization. The precursor ion and product ion were *m/z* 435.93→144.81 for rivaroxaban and *m/z* 285.1→193.1 for diazepam (internal standard, IS), respectively. The optimal MS parameters were defined as follows: the cone voltages were set at 35 V and 50 V for rivaroxaban and IS; the collision energies were set at 30 V and 25 V for rivaroxaban and IS. Masslynx 4.1 software (Waters Corp., Milford, MA, USA) was used for data acquisition, and the UPLC-MS/MS chromatogram of blank plasma that was spiked with rivaroxaban and IS was shown below (Figure 2).

### *In vivo* pharmacokinetic experiment

Ten specific pathogen-free (SPF) grade Sprague–Dawley rats (male, 220 ± 20 g) were provided by Wenzhou Medical University Laboratory Animal Research Centre. All experimental procedures and protocols were reviewed and approved by the Animal Care and Use Committee of Wenzhou Medical University and were in accordance with the Guide for the Care and Use of Laboratory Animals (ID Number: wydw2019-650). Ten rats were randomly divided into two groups: the ticagrelor group (*n* = 5) and the control group (*n* = 5). Rats were bred at 25 °C with 60 ± 5% humidity and a 12 h dark/light cycle. Enough tap water and normal chow were provided *ad libitum*. The rats were placed under the above conditions for one week before initiating the experiment.

The ticagrelor group was pre-treated with ticagrelor by oral gavage at a dose of 10 mg/kg/day (dissolved in CMC-Na solution) for two weeks before the administration of rivaroxaban. The control group also received equal amounts of the vehicle (CMC-Na solution). Rivaroxaban was dissolved in dimethyl sulfoxide (DMSO) and was then diluted with polyethylene glycol 200 so that DMSO accounted for 5% of the total volume. Each group of rats was given an oral gavage of rivaroxaban at a dose of 10 mg/kg, and then blood was collected via the rat’s tail vein for analysis at 0.083, 0.25, 0.5, 1, 2, 3, 4, 6, 8, 12 and 24 h. Samples were collected into 1.5 mL centrifuge tubes and were immediately centrifuged at 4000 rpm for 10 min.

### Plasma sample preparation

Every 100 µL of rat plasma was added with 200 µL of acetonitrile in 0.5 μg/mL of IS, followed by vortex mixing for 1 min. After centrifugation at 13000 rpm for 15 min, 5 µL of supernatant was prepared for UPLC-MS/MS system to analyse.

### Preparation of rat liver microsomes

The pooled rat liver microsomes (RLMs) were obtained from 8 rats, which were weighed and homogenized with cold 0.01 mM phosphate-buffered saline (PBS) containing 0.25 mM sucrose. After centrifuging at 11000 rpm for 15 min, the supernatants were separated and transferred into new tubes for another 15 min centrifugation at 11000 rpm. Then ultracentrifugation was performed at 10000 rpm at 4 °C for 1 h, and the microsomal pellets were resuspended with cold 0.01 mM PBS and stored at −80 °C. The protein concentration of RLMs was determined using the Bradford Protein Assay Kit (Thermo Scientific, Waltham, MA, USA).

### Incubation conditions and sample preparation

The incubation system was composed of RLMs with a concentration of 0.5 mg/mL, 100 mM potassium phosphate buffer (pH 7.4), and a series concentrations of rivaroxaban (1, 2.5, 5, 10, 25, 50 and 100 μM). The incubation time was 30 min. Incubations were initiated following a 5 min pre-incubation in a shaking water bath at 37 °C. 1 mM NADPH was added to initiate the reaction in a final volume of 200 μL. The reaction was stopped immediately by cooling to −80 °C. After that, 20 μL IS working solution and 200 μL acetonitrile were added. Centrifugation was performed at 13000 rpm for 5 min after vortexing for 1 min, and the supernatant was collected. Each incubation sample study was performed in separate EP tubes, performed in triplicate and analysed by UPLC-MS/MS.

### *In vitro* interaction studies in RLMs

Ticagrelor was applied as the inhibitor to determine the half-maximal inhibitory concentration (IC_50_). The incubation mixture in a total volume of 200 μL contained 0.5 mg/mL of RLMs, 100 mM potassium phosphate buffer (pH 7.4), rivaroxaban (20 μM), ticagrelor (1, 2.5, 5, 10, 25, 50 and 100 μM) and 1 mM NADPH. After preincubation in a shaking water bath at 37 °C for 5 min, NADPH was added to initiate the reaction in a final volume of 200 μL. The reaction was performed for 30 min and then stopped by cooling to −80 °C immediately. After that, 20 μL of IS working solution and 200 μL of acetonitrile were added. Centrifugation was performed at 13000 rpm for 5 min after vortexing for 1 min. The supernatant mixture (2 μL) was injected into the UPLC–MS/MS system for analysis.

### Statistical analysis

The GraphPad (version 8.0; GraphPad Software Inc., San Diego, CA, USA) was applied to calculate IC_50_ and plot plasma concentration-time curves. The pharmacokinetic parameters using non-compartmental analysis were calculated by DAS (version 3.2.8; Wenzhou Medical University, China). Statistical comparisons within groups were conducted by SPSS (version 25.0; SPSS Inc., Chicago, IL, USA), using Student’s *t*-test. Values of *p* < 0.05 were considered statistically significant.

## Results

### Effects of ticagrelor on the metabolism of rivaroxaban* in vivo*

As shown in Table 1, when pre-treated with ticagrelor, the *C*_max_ value of rivaroxaban was significantly increased from 221.34 ± 53.33 to 691.18 ± 238.31 ng/mL (*p* < 0.05). The AUC_(0–_*_t_*_)_ value of rivaroxaban increased from 1060.97 ± 291.21 to 3483.03 ± 753.83 μg · h/L, and the AUC_(0–∞)_ value also increased from 1065.90 ± 299.44 to 3583.33 ± 736.02 μg · h/L. All these differences were both significant (*p* < 0.05). Moreover, the MRT_(0–∞)_ of rivaroxaban increased from 4.41 ± 0.79 to 5.97 ± 1.11 h, while the intrinsic clearance decreased from 9.93 ± 2.55 to 2.89 ± 0.63 L/h/kg (both *p* < 0.05) after pre-treated with ticagrelor. There was no statistical significance of MRT_(0–_*_t_*_)_ and *T*_max_ between the two groups.

**Table 1.** The main pharmacokinetic parameters of rivaroxaban in the two groups (*n* = 5 for each group, Mean ± SD).

Pharmacokinetic parameters	unit	Rivaroxaban	Ticagrelor + rivaroxaban
AUC_(0–_*_t_*_)_	ng/mL·h	1060.97 ± 291.21	3483.03 ± 753.83*
AUC _(0–∞)_	ng/mL·h	1065.90 ± 299.44	3583.33 ± 736.02*
MRT_(0–_*_t_*_)_	h	4.32 ± 0.66	5.18 ± 0.91
MRT _(0–∞)_	h	4.41 ± 0.79	5.97 ± 1.11*
t_1/2_	h	2.64 ± 0.72	5.12 ± 1.65*
*T*_max_	h	2.00 ± 0.71	2.00 ± 0.71
Vz	L/kg	36.23 ± 5.48	21.89 ± 9.00*
CL	L/h/kg	9.93 ± 2.55	2.89 ± 0.63*
*C*_max_	ng/mL	221.34 ± 53.33	691.14 ± 238.31*

*p < 0.05 indicates significant difference between the two groups.

**Figure 3.**
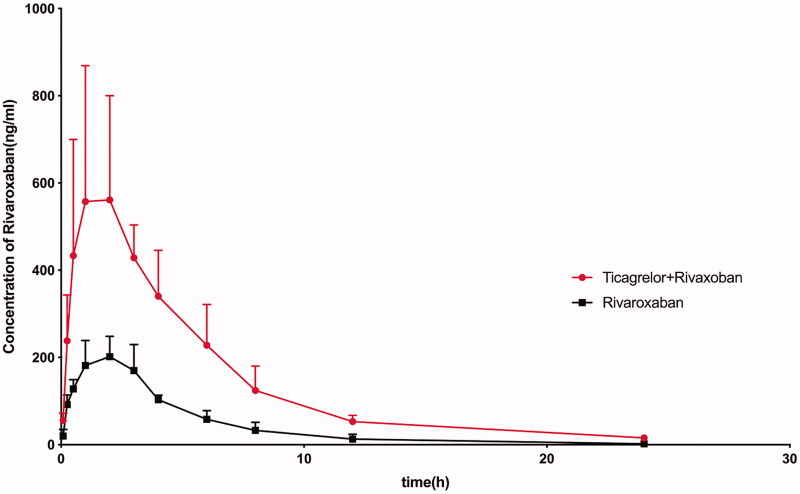
The mean plasma concentration–time curves of rivaroxaban in the experimental and control groups (*n* = 5 for each group).

The mean plasma concentration–time curves after oral administration of rivaroxaban or oral administration of rivaroxaban with the pre-treatment of ticagrelor are presented in Figure 3.

### Effects of ticagrelor on the metabolism of rivaroxaban *in vitro*

**Figure 4.**
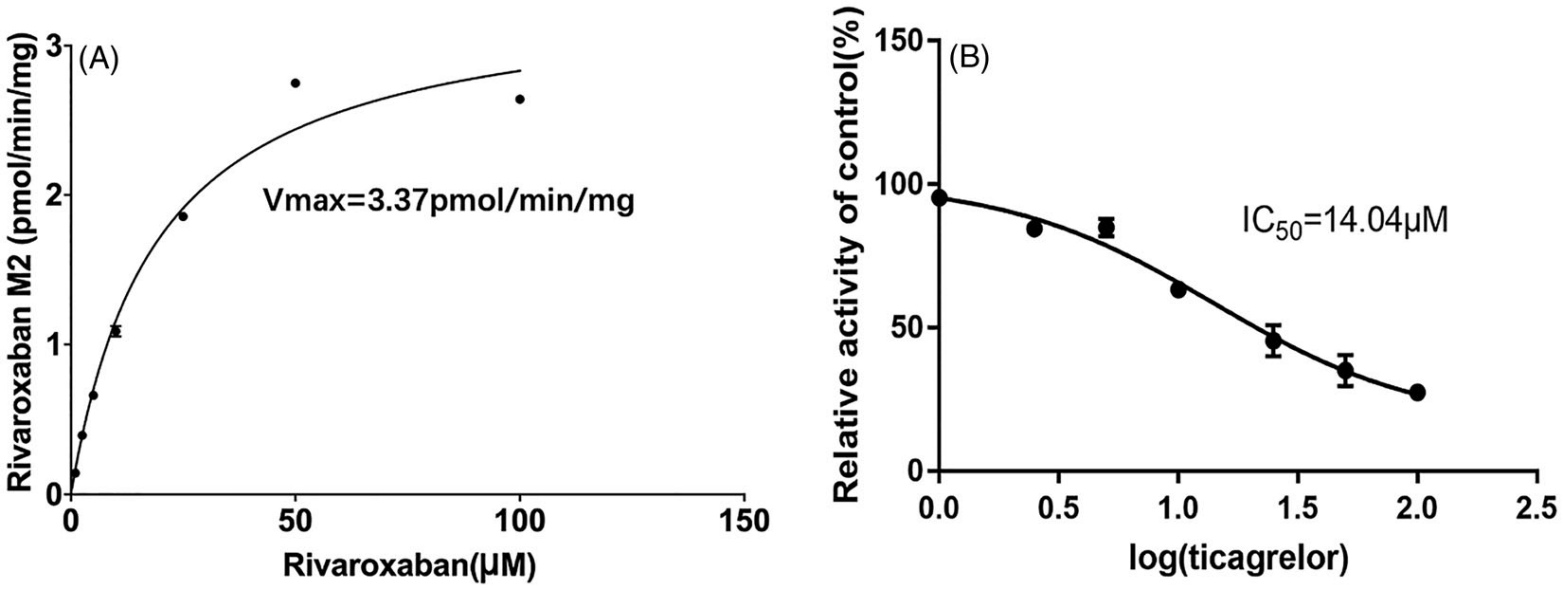
The Michaelis–Menten curve of rivaroxaban (A) and the inhibitory effect of ticagrelor on rivaroxaban metabolism (B) in RLMs. (values are Mean ± SD, *n* = 3 for each concentration of ticagrelor).

We selected 2 μL of RLMs with a concentration of 0.5 mg/mL in PBS for 30 minutes as the condition for enzyme kinetic studies. Within this condition, we determined the kinetic parameters of rivaroxaban by adding 1, 2.5, 5, 10, 25, 50 and 100 μM of ticagrelor to the incubation system in a final volume of 200 μL. The *V*_max_ and *K_m_* values for rivaroxaban were 3.37 pmol/min/mg and 19.12 μM, respectively (R squared 0.979). Following by adding ticagrelor, the metabolism of rivaroxaban was inhibited to various degrees in a dose dependent manner (Figure 4). The IC_50_ in RLMs was 14.04 μM. The results illustrated that ticagrelor can inhibit the metabolism of rivaroxaban in RLMs.

## Discussion

Our data indicated that oral administration of ticagrelor 10 mg/kg/day for 14 days could increase the AUC value of rivaroxaban by 3.3-fold and *C*_max_ value by 3.1-fold, and significantly decrease the metabolic clearance rate of rivaroxaban *in vivo*. Similarly, the *in vitro* enzyme kinetic study showed that the metabolism of rivaroxaban was inhibited by ticagrelor in a dose dependent manner with the IC_50_ value of 14.04 μM in RLMs, inferring that ticagrelor might decrease rivaroxaban metabolic clearance value by inhibiting the activity of cytochrome P450. To our knowledge, this study is the first report to investigate the effects of ticagrelor on the pharmacokinetics of rivaroxaban in rats.

Ticagrelor has been reported as a substrate and an inhibitor of CYP3A4 enzyme (Teng et al. 2010). Previous studies have shown that the ticagrelor could affect the pharmacokinetic profiles of some CYP3A4 substrates. An *in vitro* study demonstrated that ticagrelor could inhibit the 4-hydroxylation of midazolam with an IC_50_ value of 8.2 μM (Zhou et al. 2011). Oral administration of ticagrelor increased AUC_0–_*_t_*, *C*_min_, and *C*_max_ values of ethinyloestradiol by 20%, 20%, and 31%, respectively, in healthy volunteers (Butler and Teng 2011). Concomitant administration of ticagrelor with atorvastatin could increase the *C*_max_ and AUC values of atorvastatin by 23% and 36%, respectively. Co-administration of ticagrelor with simvastatin lead to the increment of *C*_max_ and AUC values of simvastatin by 81% and 56%, respectively. In some individuals, as high as 2- to 3-fold increase in AUC value of simvastatin could be observed (Teng et al. 2013). Creatine kinase elevation and rhabdomyolysis have also been reported in clinical settings (Kido et al. 2015; Beavers 2019). Since rivaroxaban is a substrate of CYP3A4, the increased systemic exposure of rivaroxaban in our study could be explained by the inhibition effect of ticagrelor on CYP3A4.

Anticoagulation in AF patients with ACS or undergoing percutaneous coronary intervention (PCI) is challenging. Conventional combination of VKAs with dual antiplatelet therapy (DATP) is associated with increased risk of serious bleeding (Hansen et al. 2010; Lamberts et al. 2012). Modified antithrombotic strategies that balancing bleeding risk and the incidence of coronary or embolic events are needed. In the PIONEER AF-PCI trial, low-dose rivaroxaban (15 mg once daily) plus a P2Y12 inhibitor or very-low-dose rivaroxaban (2.5 mg twice daily) plus dual antiplatelet therapy (DAPT) was associated with a lower risk of the first and total bleeding events as compared with standard triple therapy using dose-adjusted warfarin plus DAPT (Gibson et al. 2016; Chi et al. 2018). However, when co-administered with DAPT, even low-dose rivaroxaban (2.5 mg or 5 mg twice daily) increased the risk of major bleeding (2.1% vs. 0.6%, *p* < 0.001) and intracranial hemorrhage (0.6% vs. 0.2%, *p* = 0.009) (Mega et al. 2012). Besides, more intense antiplatelet therapy also increases bleeding risk. In PLATO study, ticagrelor was associated with increased non-CABG and non-procedure-related major bleeding (4.5% vs. 3.8%, and 3.1% vs. 2.3%, respectively) compared with clopidogrel, although fatal bleeding was low and did not differ between groups (Becker et al. 2011).

Combination of rivaroxaban with ticagrelor may cause potent antithrombotic effects. Rivaroxaban potently inhibits prothrombinase complex-bound factor Xa on the surface of activated platelets, thus inhibits thrombin generation and thrombin-induced platelet aggregation. Ticagrelor potently inhibits platelet activation, which may provide a catalytic surface for initiating and sustaining coagulation. Recently, it has been reported that rivaroxaban powerfully inhibited tissue factor-induced platelet aggregation in a concentration dependent manner and had a synergistic effect with ticagrelor (Perzborn et al. 2015). These findings may explain that very-low-dose rivaroxaban could reduce cardiovascular events in ACS patients. Our study revealed that the pharmacokinetic effects of ticagrelor on the metabolism of rivaroxaban raised rivaroxaban plasma concentration significantly in rats. The results demonstrated in our study and the pharmacodynamic interactions between rivaroxaban and ticagrelor suggest that caution should be paid when these two agents are co-administered in clinical practice, and rivaroxaban dose may need to be adjusted to minimize bleeding risk.

Our study has several limitations. First, the pharmacokinetic effects of ticagrelor on rivaroxaban we found in rats may not necessarily exist in human beings. Further studies need to be carried out to verify whether similar interactions truly apply in humans and, if so, whether these interactions have clinical significance. Second, as an explorative and preliminary study, there is no further information about what sort of drug–drug interaction between ticagrelor and rivaroxaban in the current study. More work needs to be done regarding the inhibition type and the precise mechanisms of ticagrelor on the metabolism of rivaroxaban in the future.

## Conclusions

A sensitive and reliable UPLC-MS/MS method was developed and applied for the determination of rivaroxaban concentration in rat plasma. The results shown in our study indicated that ticagrelor could significantly decrease the metabolic clearance rate of rivaroxaban in rats both *in vivo* and *in vitro*. Further studies need to be carried out to verify whether similar interactions truly apply in humans, and co-administration of these two drugs should be prescribed with caution in clinical practice.
